# Ideal models of good inpatient care for adults with intellectual disability: Lessons from England

**DOI:** 10.1177/00207640221140290

**Published:** 2022-12-04

**Authors:** Lisa Burrows, Georgia Page, Elena Plugaru, Bridie Kent, Mahesh Odiyoor, Sujeet Jaydeokar, Jonathan Williams, Kevin Elliot, Richard Laugharne, Rohit Shankar

**Affiliations:** 1 Royal Cornwall Hospitals NHS Trust, Truro, UK; 2 Faculty of Health, University of Plymouth, Plymouth, UK; 3 Cheshire and Wirral Partnership NHS Foundation Trust, Chester, UK; 4 Centre for Autism, Neuro Developmental Disorders, and Intellectual Disabilities (CANDDID), Chester, UK; 5 University of Chester, Chester, UK; 6 NHS England South-West, Bristol, UK; 7 Cornwall Intellectual Disability Equitable Research (CIDER), University of Plymouth Peninsula School of Medicine, Truro, UK; 8 Cornwall Partnership NHS Foundation Trust, Truro, UK

**Keywords:** Intellectual disability, autism, inpatient care, community care, discharge

## Abstract

**Background:**

In recent years, a significant proportion of inpatient facilities for people with intellectual disabilities and/or autism has been de-commissioned in England, This has resulted in individuals with intellectual disabilities being sent to distant hospitals far away from their families and carers leading to challenges in follow-up, community care and interventions. The impact of de-institutionalisation, has often caused patient trauma, family distress and subsequent discharge difficulties. Not every individual with intellectual disabilities and/or autism requires inpatient care but inpatient care when needed has to be local, adequate and appropriate.

**Aims:**

To evaluate current evidence of utility of inpatient models for people with intellectual disabilities and outline best clinical practice.

**Method:**

PubMed, CINAHL, EMBASE, Cochrane Library, Scopus, Web of Science were searched with key search terms. The search was conducted by the information specialist and identified abstracts screened further for inclusion criteria, methodological issues, and other appropriate characteristics. Twenty-three papers were included in the rapid review. Papers shortlisted had the inclusion criteria applied against the full text version independently by two reviewers. Disagreements regarding eligibility of studies was resolved by discussion and consensus within the project team. Key data related to in-patient models of care was extracted from the included papers, which included year of study, design, study objectives, target population, method/s tested, outcomes reported, country of study/studies, and results. Data extraction was performed by two reviewers and reviewed by the project team.

**Results:**

From the review of services for people with intellectual disabilities, we came across four broad models/frameworks/approaches. Evidence on what worked for inpatient service provision tended to be based on models developed and implemented locally.

**Conclusions:**

We make recommendations for the best clinical practice and standards. Both clinical service providers and policymakers need to be aware of specific needs of individuals with intellectual disability and/or autism.

## Introduction

In the past known as mental retardation, mental handicap, learning disability and now intellectual disability, as a sub-speciality of clinical psychiatry brings with it many challenges in management both at an inpatient and community levels. Intellectual disability (ID) is defined as impaired intelligence contributing to compromised social functioning that develops before adulthood and has a lasting effect on development and functioning in specific spheres. Neurodevelopmental disorders, particularly autism, have a significant overlap with ID. Thus, even though ID and autism are distinct clinical entities, for service policy, clinical provision, and care pathways in England the two conditions are often clustered together. It needs to be emphasised that there are indeed important differences with regard to the service needs of people with ID as opposed to people with autism and ideally would benefit from distinct service designs.

Mental health and behavioural issues are common co-morbidities for People with ID and/or Autism (PwID/A) (Cooper et al., 2007). The associated co-morbidities and general poorer health often results in complex clinical presentations. These are accentuated by the varying difficulties in communication with the assessors and clinicians and the different severities of disability that PwID/A present with. The complex presentations of mental distress means that PwID/A may require specialist services equipped with qualified and experienced staff that can provide the appropriate and effective assessment and care, especially at times of crisis (Devapriam et al., 2015; Purandare & Gravestock, 2019) which can be frequent.

Over the past few decades, England, has undergone significant transformation to the way that mental healthcare is provided to PwID/A. The public scrutiny of specialist inpatient psychiatric units for PwID/A that occurred after the Winterbourne View Scandal in 2011 was a catalyst for a major review of services for PwID/A leading to the Transforming Care report (Department of Health [DOH], 2012). This focused on enabling PwID/A in specialist inpatient psychiatric settings to move back into the community. The use of generic psychiatric inpatient settings for this group was emphasised whilst also endeavouring to raise the quality standards for new admissions to specialist settings (Cooper et al., 2007; DOH, 2012). The number of specialist mental health beds commissioned by Clinical Commissioning Groups (bodies which commission services at local levels though often there are nationally recommended standards) subsequently reduced by 4.5% by the end of 2015. This brought the reduction in specialist inpatient psychiatric provisions for PwID/A to almost 90% in the NHS since 1987 (Devapriam et al., 2015; Public Health England, 2016).

Whilst most PwID/A live independently, just over a fifth (21%) still require regular contact with specialist services (Devapriam, Alexander et al., 2014). Most of their psychiatric care is now through community specialist psychiatric services and local general psychiatric inpatient facilities. However, there is a continued need for specialist inpatient psychiatric resources for PwID/A. In a survey of consultant psychiatrists working with PwID/A in England (*n* = 65), 82% reported utilising specialist inpatient services on occasion for better management of mental health and/or behavioural needs of some of their patients (Guinn et al., 2016). This underlines the limited but important role of effective specialist inpatient care in the management of PwID/A.

There, remains a strong political and public focus on the need to reduce specialised psychiatric units for PwID/A but this issue is nuanced and complex. In providing clinical services, key challenges include a lack of an evidence base of effectiveness of interventions, variable service quality and geographic spread of specialist psychiatric units, compounded further by a lack of suitable community capability to support the needs of this vulnerable population (Abraham et al., 2022; Jones et al., 2021). Currently there are too few beds in certain regions of England resulting in people being admitted out of their local region. These admissions far from home do not facilitate effective treatment and timely and successful discharge (Abraham et al., 2022). Work needs to be done to improve inpatient service provision for PwID/A, particularly defining what should be offered and how. In view of these observations about variability of access to appropriate services, we decided to explore community and inpatient services for PwID/A.

A systematic review recently considered the likely effectiveness of in-patient treatment for this population to compare and contrast differing models of in-patient care (Melvin et al., 2022). We sought to analyse the existing evidence on how to provide specialist psychiatric inpatient services for this group of patients in order to inform best practice. A rapid review of the literature was conducted to identify the key evidence for the analysis.

## Methodology

### Scope

The phenomenon of interest is the model of care for non- forensic inpatient services for people with ID/A and/or a mental health condition/behaviour that challenge. The range of study designs included ranged from RCT through to published case study in peer reviewed index linked journals. Studies conducted within United Kingdom and from other countries with similar characteristics to the UK where these are robust in design and content were also included. Dates were limited to 2010 onwards to identify the most up-to-date evidence; only studies published in English were considered.

### Searching

Databases included PubMed, CINAHL, EMBASE, Cochrane Library, Scopus, Web of Science. The search was conducted by the information specialist. About 3,980 references were identified and added to an EndNote library.

### Title and abstract screening

Before starting the title and abstract screening, 1,277 duplicates were removed from the working reference list, which brought the number down to 3,210 references. The screened according to the selection criteria was completed by three reviewers, process which concluded with the identification of 238 references. The list was then shared with another two reviewers from the team who undertook another screening. Rayyan was used during this screening stage.

### Full text screening

Fifty-five references were included in the full text screening, and 3 were considered as potentially relevant. The full text of these references, including the potentially relevant ones, was retrieved for closer examination. All 58 articles were exported from Rayyan to Excel. Only the 34 included articles were put into the data extraction table. Whilst going through these articles for data extraction, 11 further articles were removed for methodological reasons. These 11 articles were either reviews, descriptive research papers or book chapters and as per the inclusion criteria in the protocol, we are looking for peer reviewed studies ranging from Randomised Control Trials (RCTs) to case studies between 2010 and 2021.

As a result, 23 papers were to be included in the rapid review. The 35 papers excluded fell into the following exclusion reasons: (14 excluded based on inclusion criteria, 9 were excluded as these were conference abstracts and no full text was found, 1 was in German, 11 were excluded due to methodological reasons).

We erred on the side of inclusion if there was any doubt about its inclusion to ensure no potentially relevant papers were missed. The inclusion criteria were then applied against the full text version of the papers independently by two reviewers. Disagreements regarding eligibility of studies was resolved by discussion and consensus within the project team.

Key data related to in-patient models of care was extracted from the included papers, which included year of study, design, study objectives, target population, method/s tested, outcomes reported, country of study/studies, and results. Data extraction was performed by two reviewers and reviewed by the project team.

## Results and findings

### Models described for inpatient service provision

Evidence on what worked for inpatient service provision tended to be based on models developed and implemented locally. From the review of services, we came across four broad models/frameworks/approaches (Bartle et al., 2016; Devapriam, Alexander et al., 2014; Marsden & Giles, 2017; Richings et al., 2011). Table 1 presents the four models describing the key elements and setting and also providing a critical opinion and analysis of these models. However, there is a dearth of evidence describing clear outcomes of different models of care and no comparison trials.

**Table 1.** Description and critical opinion of inpatient models/approaches for PwID/A.

Model	Key elements	Setting	Critical opinion
**The Care-pathways model** ‘*Impact of care pathway-based approach on outcomes in a specialist intellectual disability inpatient unit*’ (Devapriam, Alexander et al., 2014).	1. Streamlining of the processes with clear timeframes and documentation of milestones and clinical interventions resulting in a reduction in unnecessary delays. 2. Early-stage discharge planning. 3. Multi-disciplinary Team (MDT) working and care planning.	Specialist inpatient unit for PwID/A in Leicester, England which has eight assessment and treatment beds.	This approach focusses on the processes of inpatient care and not about the therapeutic aspect of care. By focussing on the process, this approach showcases certain aspects of inpatient care. It highlights evaluation, timely assessments and treatments. Continuity of care has an impact on the length of stay.
**BCATS model** ‘*Service evaluation of an integrated assessment and treatment service for people with intellectual disability with behavioural and mental health problems*’ (Richings et al., 2011)	1. Proactive community MDT input prior to inpatient admission. 2. Clear framework for pre-admission input. 3. Scope for day assessment before considering admission. 4. Holistic assessment and MDT working.	Birmingham Community Assessment and Treatment service in England integrates assertive outreach, day assessments and inpatient beds and has 6 inpatient beds. The BCATs model specifies which assessments should be carried out, the frequency of assessments and MDT reviews, how outcomes should be measured and emphasises enhanced response to service users in crisis.	This model seems to be a precursor to Intensive Support method post Transforming Care and seems to be closely linked with enhanced Community Learning Disability Team approach. A positive of this model is that it views inpatient care in the context of what is happening in the community. However, not much is said about discharge such as what happens after discharge, what is done to facilitate discharge and whether the BCATs service stays involved post discharge particularly with a focus on rehabilitation. There is no clear indication of whether this model is still being employed or if it has moved to the now more common Intensive Support approach.
***Planning Live*** *‘Planning Live’: using a person-centred intervention to reduce admissions to and length of stay in learning disability inpatient facilities* (Bartle et al., 2016).	1. The ‘Planning Live’ process involves family and carers from the onset as valued members of the MDT, and it aims to gather knowledge about a person from different sources, identify gaps in understanding to identify areas of improvement to formulate an action (care) plan to support the PwID/A in the wider system/network. 2. The essential components are multi-stakeholder meetings which help prevent inpatient admission for people referred to inpatient services, as well as contribute to a significant reduction in the length of admission in inpatient units.	102 clients were referred for a possible inpatient admission between April 2013 and April 2015 and took part in the ‘Planning Live’ process. The meetings facilitated in a relaxed and open style, everyone involved in the care and support of the PwID/A were welcome to join these meetings.	The model resulted in a reduction of inpatient duration but led to increased number of hospital admissions. This has been viewed as a possible failure. However, it could be that the model helped expose the unmet need in community. As long as admissions are appropriate, goal focused and assisted with timely discharge it should not be thought to be a failure. The enhanced engagement with multi-agency stakeholders is to a degree reciprocated in the current Care and Treatment Reviews (CTRs). Where CTRs differ from Planning Live is that CTR are advisory and a body to oversee and recommend best practice. Planning Live was delivered by stakeholders directly responsible for the individual PwID/A and thus ‘owned’ the concern.
***4C framework*** *The 4C framework for making reasonable adjustments for people with learning disabilities* (Marsden & Giles, 2017)	The paper identified 4 key themes as part of the framework. Changes to these key themes were thought to assist ward staff in identifying and making reasonable adjustments to the care of PwID/A. This framework supports staff deliver person-centred, safe and effective care. The themes are: 1. Communication – finding ways to better understand and communicate with PwID/A, who might have communication impairments. 2. Choice-making – this theme highlights the importance of staff being familiar with the laws related to consent and choice-making. The framework draws attention the issue of mental capacity, informed choice making and offers tools on supporting staff with navigating this. 3. Collaboration – encouraged between various players in the care of the PwID/A, such as family and friends, therapists, community nurses, social care workers etc. 4. Coordination – highlights the importance of coordinating care for PwID/A at every care level.	The authors attended ward manager and matron group meetings to collect their claims, concerns and issues, then conducted a collaborative thematic analysis with the group members to identify the main themes.	The article discussed the 4C framework and provided guidance on how to make reasonable adjustments for PwID/A in hospital. Implementation and evidence of whether this framework improves hospital care delivery to PwID/A is not discussed or presented. Further, the focus is on the inpatient setting and not on prevention or rehabilitation. It is acknowledged that the workplace is often the best learning resource and environment for healthcare professionals.

Quality evaluators tend to adopt variations of these four models. The Royal College of Psychiatrists Quality Network for Learning Disability Services (QNLD) framework is a Care Pathways model where the focus is on quality and care delivery methods (Devapriam, Alexander et al., 2014). Intensive support teams (ISTs), which are multidisciplinary specialist teams that have for many years been advocated as the best services to help PwID/A and challenging behaviour remain within their local communities, has commonalities with a BCATS model (Richings et al., 2011). Care and Treatment Reviews (CTRs), NHS England’s structure used by commissioners for PwID/A to improve the quality-of-care people receive in hospital, are similar to the Planning Live and 4C models (Bartle et al., 2016; Marsden & Giles, 2017).

## Discussion and analysis

### Good clinical outcomes

There is clear evidence from 168 PwID/A admitted over 8 years to a single specialist unit in London that admission benefits patients in reducing their cause of distress such as mental illness, challenging behaviour and/or suffering due to their autism (8). The mean change in patients’ scores on the Health of the Nations Scale (HoNOS-LD), a validated psychiatric outcome tool, was 18 points, from a mean of 32 on admission to a mean of 14 on discharge. However,this positive reduction was achieved months before actual discharge. This may indicate that post-discharge options may be difficult to obtain and place patients.

### Positive inpatient experiences

Not surprisingly, PwID/A placed in out of area facilities reported a strain on relationships and contact with their family and friends and limited opportunities for them to practice and develop their cultural and religious identities, especially for ethnic minorities. They also reported negative treatment from staff and discomforting environments (Chinn et al., 2011).

Relationships and experiences with staff were very important for PwID/A to feel safe, with one study reporting the need for staff to be trustworthy, consistent and sensitive in times of distress and uncertainty (Claire Lloyd et al., 2013). Service users also appreciated the range of different staff personalities and care-styles, acknowledging there is no ‘right way’ for staff to interact (Claire Lloyd et al., 2013).

For PwID/A three key areas in their mental health were identified as important in inpatient care: avoiding boredom, managing their emotional responses through good communication and considering their safety and risk management (Taua et al., 2015). For some, the use of seclusion is stressful, whereas others found it useful in calming them down. PwID/A expressed a need for alternatives to seclusion and the use of medication, including de-escalation through dialogue (Roscoe et al., 2016). This highlights an important point in the wider context of segregation or modified low sensory environments for PwID/A although this has not been little explored in research but is currently being debated nationally (Jones et al., 2021).

The experiences of PwID/A, their main carers and the service providers in general psychiatric units was examined (Donner et al., 2010). Service users’ experiences of generic psychiatric inpatient services were predominantly negative with feelings of disempowerment, lack of control, being unoccupied most of the time and perceived huge barriers to accessing help in the first place. Feedback from carers suggested local admissions gave them important respite. Service providers identified significant difficulties including challenges in communication with PwID/A, difficulties of joint working between local ID and mental health community services and limited implementation of national policies. These findings pre-date the Transforming Care agenda (DOH, 2012). The guidance came to being post Winterbourne to supports NHS and local authority commissioners in England reduce the number of PwID/A in mental health inpatient settings and to develop community alternatives to inpatient care. However, they highlight the need to ensure robust implementation of strategies to improve local mental health services so that it is effective in supporting PwID/A and the need to recognise the complexity of this group in generalist settings (NDTi, 2017)

### Positive staff experience

Research on staff experiences particularly focused on supporting families and carers. Professionals in specialist psychiatric units understood the impact of stress and additional needs of PwID/A and their carers during the admission (James, 2016). They identified the need for effective communication as a key facilitator to support PwID and their families/carers. However, this was impacted by the limitations of resource and time availability. Not surprisingly, it was identified that professional relationships had a profound effect on the way families/carers appreciated their value and their own sense of self.

### Appropriate staff training

One study explored adaptations in inpatient psychiatric services made to meet autism needs (Jones et al., 2021). It looked at clinical skills, current length of stay and use of restrictive interventions for people with autism. A lack of professional competencies, training and skills in autism across staff including psychiatrists, especially in general mental health services, lead to inconsistent and variable care. This highlights the need for a standard training framework for professionals with a stratified approach to provide a range of training measures given the multidisciplinary nature of inpatient care delivery. The forthcoming autism training developed by Health Education England might address some of these issues (Health Education England, 2022).

### Adapted management modalities

Effective communication was identified by service users as an effective strategy to help them manage their emotional responses (Taua et al., 2015). Communication/talking as a key therapeutic response was suggested by several service users as an important alternative to seclusion and (increased) medication use. Based on this, these authors recommend a key strategy called ‘Focused Interaction’ which requires professionals, particularly nurses, to explore desired ways of coping with s emotions of service users and being creative in negotiating individual’s coping, making sure there is two-way communication. In individuals with moderate to severe PwID/A whose social behaviours may be limited, these authors emphasise the importance of building a rapport and a relationship. Overall, this finding has demonstrated that structured talking has a role in de-escalation similar to low stimulus environment and distraction behavioural strategies. Communication was also found to be important in carer-professional relationships to support provision (James, 2016).

There is significant lack of published research into therapy for PwID/A in non-forensic specialist inpatient setting. However, the limited research available suggests that specific PwID/A can find certain therapies useful and beneficial, with some stating it developed their communication and negotiation skills thus making them feel valued and understood as individuals [16] who have specific needs. There is generalisable evidence which needs considering for providing any therapy for PwID/A (Roscoe et al., 2016).

### Delays in discharge due to problems with community living

Instances have been described when delayed discharge was due to a systems issue in the discharge process such as no suitable placements being identified or available, ineffective communication with the community services or local authorities and lack of a rehabilitation framework (Devapriam, Alexander et al., 2014; Devapriam, Gangadharan et al., 2014; Odiyoor et al., 2019; Richings et al., 2011; Washington et al., 2019). One study found that in 83% of cases, the cause of delayed discharge was finding a new or alternative care provider, placement, accommodation or funder (Washington et al., 2019). A quarter of people in NHS assessment and treatment units had finished their treatment but there were no plans for discharge (Mansell et al., 2010). Similar findings were highlighted in another study which found a statistical difference in time to finish treatment to time to discharge, irrespective of neurodevelopmental, mental or physical health co-morbidity (Abraham et al., 2022).

### Care away from home

The negative impact on inpatients receiving care far away from home is likely to include feelings of abandonment, degree of helplessness, and confusion, putting an additional strain on relationships with family members and wider social networks (McKinstry et al., 2010). Similarly, distant hospital admissions reduced the access to and availability of therapeutic and educational activities after discharge.

### Consistent treatment models

A significant barrier is the lack of understanding of broader care models which extend from inpatient settings to the local community. There needs to be appropriate support and services developed to facilitate this engagement (Washington et al., 2019).

### Other issues

There are certain other concerns which have not been explored scientifically need mentioning. Political thinking, particularly led by mainstream media, mostly using case examples of poor practice have promoted closure of specialised psychiatric hospitals for PwID/A. In recent non- mental health medical scandals such as the Mid Staffs (where an estimated 1,200 patients died as a result of poor care at a general hospital in Staffordshire, England between 2006 to 2014), the focus was rightly on improving care, developing better care models and building public trust but not closing down general physical health beds or hospitals (The Guardian, 2013). Similarly, the political focus on inpatient settings for PwID/A without due attribution of the need to create capable local communities by tackling issues such as housing, care provider competences, recruitment etc. has perpetuated for some a culture of inpatient dependence and institutionalisation.

## Developing a good model of inpatient care

### Individuals

#### The opinions and views of PwID/A

Coproduction with services users is the key in identifying what works and does not work in inpatient service provision as patients can identify areas where their needs are important, describe what they value and what makes them feel safe in inpatient care and suggest alternatives to seclusion and medication. The evidence described suggests that they value highly degree of good communication with staff.

#### Holistic care

Admission, re-admission, discharge, and rehabilitation are themes that were discussed by several authors within the wider service model for PwID/A. Additionally, it is encouraged to gather a holistic understanding of the PwID/A to develop a clear and thorough action plan. More specifically, clinical processes should be implemented using person-centred- planning tools, at various points during an admission particularly at the early stages of that admission taking into account issues of mental capacity, consent and advocacy. Meaningful involvement of a wide range of stakeholders appropriate to the individual case is recommended. Other identified issues for inpatient settings to address include the prevention of boredom, staff patient relationships, patient disempowerment, a better physical environment, effective communication, and providing person centred risk management strategies.

### Services

#### The role of mainstream psychiatric inpatient services

There is a role for mainstream services to provide inpatient care for PwID/A. Generic inpatient admissions are not always negatively perceived when used at the right time, for the right reasons. However negative patient and provider experiences suggest they need to be person centred and specialised to the population group. Staff training to augment their skills and knowledge is critical in enhancing this care model. There needs to be suitable guidance centred around presenting clinical complexity, local resource availability and staff competency to explore and make swift proactive decisions, preferably with patient’s family, carers and/or advocate, as to whether mainstream psychiatric admission is in the person’s best interest. Good links, pathways of care with local ID specialist services including intensive support and crisis support services is essential in supporting repatriation swiftly from generalist inpatient care.

It is recognised clinically that service provisions for autistic people without ID while overlapping significantly need to be distinct to those with ID or only PwID (Jones et al., 2021). However, the needs of PwID and autistic people have been condensed together by national initiatives such as the Transforming Care Agenda (DOH, 2012). This has led to confusion and lack of clarity in care delivery. Unfortunately, available evidence is not clear on what specific and different approaches the two sub-populations could benefit from and further research is need.

### Policy

#### Appropriate staff training

Staffing practices and training are important aspects of care. There needs to be a recognition of the importance of qualified staff (particularly nurses) in the care of PwID/A. These should be addressed when planning services. In addition, there is a need for ongoing staged training for autism, reasonable adjustments for ID, communication training, consistency of approach, and training on legal aspects including the Mental Capacity Act. Therapeutic approaches including positive behaviour support and a sensory environment needs to be seen as essential components for specialist inpatient units.

#### Location of in-patient placements

Policy makers are urged to consider the perspective of PwID/A with complex mental health needs and take seriously the drawbacks in placing individuals out of their local area. There is a role for shorter stay assessment and treatment units and longer stay complex continuing care and rehabilitation in their local community.

#### Broader issues in relation to delay of discharge

A good model of effective inpatient care needs to incorporate an equal focus on step-down services as to those aimed at reducing admissions. The focus on effective and timely discharge requires a multi-agency approach to strategic commissioning and planning.

## Conclusion

Inpatient care must be integrated with community services with discharge planning from the start of an admission. Models of care need to be described, evaluated and must emphasise the importance of positive communication between, patients, staff and carers. The flow of care for the patient, from admission to discharge, needs to be proactive and dynamic, but is frequently delayed and is worse when admited to distant units.

The need and demand for inpatient care for PwID/A persists and must be met in a careful appropriate manner. The response to bad inpatient care may be to develop, research and implement models of good inpatient care rather than demand the closure of inpatient ID facilities. The ‘cure’ for atrocities on PwID/A should not be worse than the ‘disease’. Inpatient units in turn should openly describe their care model and measure clinical, and patient reported outcomes as routine. PwID/A needing inpatient support can be broadly divided into three. These include those who can access mainstream facilities with or without reasonable adjustments and those requiring specialised psychiatric care. The role of each of these therapy systems needs clear and targeted defining to identify what specific needs are met best in which setting.
